# A qualitative exploration of factors that influence engagement with a digital mental health intervention for women with metastatic breast cancer: Finding My Way-Advanced

**DOI:** 10.1007/s00520-025-09379-9

**Published:** 2025-03-31

**Authors:** Stephanie Bourboulis, Emma Kemp, the Finding My Way-Advanced Authorship Group, Lisa Beatty

**Affiliations:** 1 Flinders University, College of Education, Psychology & Social Work, Flinders University Institute of Mental Health & Wellbeing, Adelaide, SA Australia; 2 Flinders University, College of Medicine and Public Health, Flinders Health and Medical Research Institute, GPO Box 2100, Adelaide, SA 5001 Australia

**Keywords:** Metastatic breast cancer, Engagement, Facilitators, Barriers, Digital mental health intervention

## Abstract

**Purpose:**

While digital mental health interventions (DMHI) may improve access to timely support for women with metastatic breast cancer (MBC), scant research exists on how metastatic survivors engage with these programs and what factors impact usage. This study therefore qualitatively explored barriers and facilitators influencing engagement with (a) digital resources (e.g., information websites) generally and (b) in particular, *Finding My Way-Advanced* (*FMW-A*), an interactive self-directed DMHI containing psychoeducation, therapeutic activities, and multimedia content tailored for women with MBC.

**Methods:**

Twenty women with MBC, who received either the FMW-A 6-week intervention (*n* = 13) or a digital-resource control (*n* = 7) as part of a larger RCT, participated in semi-structured interviews. Transcripts were coded using framework analysis against three domains of Bowen’s feasibility framework (acceptability, demand, practicality).

**Results:**

Engagement was high among intervention participants overall (six modules completed *n* = 3; five *n* = 5; four *n* = 1; two *n* = 4). Five overarching themes were identified. Three a priori domains related to FMW-A have high *acceptability* and *practicality*, but some *demand* barriers. Two inductively derived themes related to varied perceptions of *navigability/layout* and *deriving personal benefits/impact*. Key subtheme facilitators were program *satisfaction*, *convenience*, experiencing *personal benefits/impact*, and *ease of navigation*. Subtheme barriers were *technical access barriers*, experiencing *time burden*, and *suboptimal intervention timing* relative to time since diagnosis.

**Conclusions:**

This study confirms that while many DMHI facilitators and barriers are consistent with those identified in curatively treated settings, some factors become more salient in metastatic populations (e.g., time burden). This research also offers novel insights that deriving early personal benefit promotes engagement and provides targets for future program improvements to address navigation and optimal-timing challenges.

## Introduction

Despite improved treatments and survival duration (1, 2), metastatic breast cancer (MBC) remains incurable and brings significant challenges, including high physical symptom burden (e.g., pain and fatigue), impaired health-related quality of life, clinically elevated distress (occurring in 35–43% of women), fear of cancer progression, and existential difficulties including heightened awareness of eventual mortality (2–4). Yet few psychotherapeutic resources have been developed specifically to address these challenges, and many women with MBC continue to report unmet needs (5). Although face-to-face interventions are available, much of the current evidence base is derived from early-stage breast cancer (6). Further, under half of cancer patients take up these interventions (7), due to personal, illness-related, cost, and geographic uptake barriers (e.g., travel distance) (8). Two systematic reviews documented that (a) few interventions have been developed specifically for MBC (9) and (b), of those that have, interventions with the strongest evidence—group-based psychological therapy—also have the highest participation burden and lowest uptake/engagement (10).

One commonly hailed solution to these challenges is online delivery. Given increasing evidence in early-stage cancer populations for reducing distress (11, 12), depression, anxiety (13), and fear of recurrence (14), digital mental health interventions (DMHI) hold promise for translation to the metastatic setting, with strong demand and interest demonstrated from women with MBC (15, 16). Indeed, there may be unique applicability, given DMHI offer potential ease of access, privacy, and cost-effectiveness as motivators (5, 7). Our group recently co-designed *Finding My Way-Advanced* (*FMW-A*) (5, 17), an online program for MBC adapted from an evidence-based 6-week/6-module DMHI for individuals with early-stage cancer (8), and published the first pilot randomised controlled trial (RCT) where results confirmed feasibility and justified a definitive trial (18). Efficacy is currently being trialled (19), with an important component of evaluation being the participants’ *engagement* level, as low engagement is a known limitation of digital interventions (20, 21). Given engagement is an important predictor of treatment outcomes (22), exploring patterns of—and factors impacting—engagement is important, to ensure interventions are continually refined and tailored to improve efficacy.

Digital health engagement is a complex construct and can be measured through several indicators, with the number of modules completed being one of the most reliable (23). Of the studies investigating factors influencing engagement in curatively treated cancer survivors, the most prevalent barriers reported are *illness-related* (e.g., treatment side effects) and *intervention* (e.g., timing of intervention) (24). Specifically, those who received access towards the end of treatment expressed a preference for access upon initial diagnosis (24, 25). *Social barriers* including lack of support, low self-discipline, and importance of engaging with relatable survivors were also observed (24, 25). Less frequently reported barriers include *psychological* (e.g., avoidance, coping well), *computer-related* (e.g., no access), and *personal* (e.g., lack of time, forgetting) (24–26). On the other hand, facilitators comprise *intervention* (e.g., program satisfaction, relevant content, ease of use, ability to self-pace) (24, 27, 28), *psychological* (e.g., positive expectations of treatment, adequate social support) (26, 29), and convenience of the online format. Whether similar factors apply to metastatic populations, and/or novel factors emerge, has not been reported.

Given our group is among the first to develop a tailored DMHI for women with MBC, we aimed to qualitatively explore barriers and facilitators influencing engagement with *FMW-A*. We compared the experiences of control-group participants (who had access to a publicly available digital information-resource) with those of intervention participants, enabling us to differentiate facilitators and barriers of accessing *any* digital resource from factors unique to interactive DMHIs such as FMW-A.

## Methods

The multisite RCT full protocol has been published (19). The following methods relate specifically to the qualitative sub-study.

### Participants

Participants were females aged 18 years or over, diagnosed with MBC, who had enrolled in the *FMW-A* clinical trial (ethics approval (HREC: 2021/HRE00407)), and completed the intervention period (10 weeks post-baseline). Consecutive participants from both intervention and control groups were contacted via telephone, SMS, or email a maximum of three times and invited to complete a telephone interview. Recruitment occurred between August and November 2022, until data saturation was achieved.

### Intervention conditions

*FMW-A* is a 6-module CBT-based DMHI covering: “Navigating Healthcare,” “The Unique Challenges,” “Physical Symptoms,” “Emotional Distress,” “How You See Yourself,” and “Your Family and Friends.” Each module provides (i) psychoeducation, (ii) personal accounts from MBC survivors, and (iii) relevant CBT-based, relaxation/mindfulness, and expressive-writing worksheets. The program contains links to other reputable cancer organisations, including those provided to the control-group outlined below. Modules were released simultaneously; however, participants were encouraged to prioritise modules of relevance, self-pace, and use the program over the 6-week intervention period.

Control-group participants were provided access to Breast Cancer Network Australia’s (BCNA’s) online resource, ‘*My Journey’*, which can be tailored for MBC, and covers a range of topics (e.g., emotional wellbeing, treatments, side effects). More information can be found in the published protocol (19).

### Procedure

Consenting participants were provided with a topic guide (Table 1) prior to the interview. Interviews were conducted by one author (SB), a master’s level psychology student under the supervision of senior researchers experienced in qualitative methodology (LB, EK). Interviews were audio recorded, transcribed verbatim, and coded. Saturation was determined through an iterative process, comparing new data with previously collected data after each interview; after completing two consecutive (18th–19th) interviews with no new data emerging, content saturation was deemed to have occurred. Due to the high information power of the study population, saturation was able to be achieved with fewer interviews (30).

**Table 1:** Topic guide domains

Topic area	Questions
Overall adherence	• How much of the program/resource did you use overall/complete? • In general, what influenced your stopping to use/completing the program/resource?
Intervention factors	• Overall, how would you describe your satisfaction with the program/online resource? • Were there any aspects of the program/resource that made you feel more or less inclined to use it? • Specifically, did you find the information to be relevant, interesting, and of good quality? • How easy/difficult was it to read/understand the information in the program/resource? • Did your level of satisfaction with the information influence how much you used the program/resource? • What did you think of the interface and structure of the program/resource? • Where there any particular modules/parts of the resource that you liked or disliked compared to other modules/parts of the resource? • *Intervention group only:* Were there particular worksheets that you liked or disliked using? What did you like/not like about them? • *Intervention group only:* How easy or difficult to understand were the directions on how to complete the worksheets/activities? • Was the program/resource difficult to use in any way? If so, in what ways was it difficult? • How convenient did you find the program/resource to use? What aspects made it convenient/inconvenient?
Internet factors	• Did you have any difficulties with accessing or using a device (e.g., phone, tablet, laptop or other computer) or the internet? *Prompt:* Did you have difficulty with (a) accessing a suitable device to use the program/resources (b) accessing reliable/affordable internet or data to use the program/resources (c) enrolling in the study or logging into the program/navigating to the resource (d) navigating around the different parts of the program/resource (e) concerns about privacy or internet/data security when using the resource? • (*Program group only*): How did you feel about the amount of time required to complete o Each module? o The whole program? • Overall, how did you find the online self-help format of the program/resource?
Personal factors	• Was there anything relating to your personal life or circumstances that made it difficult for you to use the program/resource? *Prompt: timing of the study/medical appointments/events/work commitments/financial stresses/other events or circumstances?* • What things, either personal or relating to the program/resource, might have increased your use of the program/resource?
General feedback	• Would you use a program/resource like this again? • Did you intend to use the program/resource more than you actually did? • Overall, did you feel that the program was worthwhile, and did it help you? • On a scale of 1 to 10, how would you rate your experience of the program/resource? • What things contributed to you giving the program/resource that rating? • Do you have any other comments or things you would like to share about your experience with the Finding My Way-Advanced program/Breast Cancer Network Australia online resource?

### Analysis

Participant’s sociodemographic, clinical characteristics and program engagement were analysed quantitatively and compared to the overall RCT sample for representativeness. Qualitative data were analysed using NVivo, guided by Braun and Clarke’s (31) six stages. Two authors (SB and EK) collaboratively coded two transcripts, to develop a mutually acceptable coding matrix, and manage reflexivity. SB then completed coding for the remaining 18 transcripts. Codes were collated into themes and subthemes using framework analysis, enabling both deductive (a priori theory-informed themes) and inductive (data-derived themes) analyses (32). For deductive analysis, three of Bowen et al.’s (33) feasibility study focus areas were selected as a priori domains: acceptability, practicality, and demand. Additional themes were simultaneously inductively developed. Consistent with our prior analyses of engagement/adherence (8, 20, 24), the frequency of emerging themes was summarised by participant’s engagement level (high vs low), to enrich the depth of qualitative analysis and establish how generalisable findings might be. Groups were operationalised by the number of modules completed, such that participants who accessed four or more modules were defined as “high-engagers,” as this met prior definitions of receiving a therapeutic dose (8, 20). Quality of resulting themes was further tested using a thematic map, which was presented to the full authorship team for validation, along with a table of finalised definitions for each theme. Following this, a meeting was held between SB and EK to finalise themes.

## Results

### Participants

Of 26 approached individuals, 23 consented and 20 were interviewed (*n* = 13 intervention; *n* = 7 control). Three participants were unable to be contacted at their interview time (*n* = 1 deceased, *n* = 2 cancelled/other commitments). Interviews lasted 17 min on average (range 8–39 min). Table 2 summarises participant characteristics and program engagement.

**Table 2:** Characteristics of interview participants

Demographic characteristic	Intervention (*n* = 13) 65%	Control (*n* = 7) 35%
Age (years) M (SD)	55 (8.2)	63 (9.2)
Married/partnered	11 (84.6%)	4 (57.1%)
Dependent children	0 (61.5%)	0 (85.7%)
Tertiary education	7 (53.9%)	4 (57.1%)
Employed	8 (61.5%)	3 (42.9%)
Ethnicity Australian Other^a^	12 (92.3%) 1 (7.7%)	6 (85.7%) 1 (14.3%)
Time since diagnosis *M* (SD) (months)	28.9 (13.7)	44.9 (42.9)
Treatments received Surgery Chemotherapy Radiotherapy Hormonal therapy Immunotherapy Other	3 (23.1%) 8 (61.5%) 8 (61.5%) 9 (69.2%) 4 (30.8%) 2 (15.4%)	3 (42.9%) 3 (42.9%) 4 (57.1%) 4 (57.1%) 0 (0%) 1 (14.3%)
De novo diagnosis (Yes)	5 (38.5%)	2 (28.6%)
Modules completed *M* (SD)	4.2 (1.6)	N/A

^a^Other includes 1 × other Caucasian and 1 × Indian

### Engagement rates

Across intervention participants, the mean number of modules completed was 4.23. Nine (69%) were considered high-engagers (completing 4 or more modules). Sub-study engagement rates were higher than the larger RCT to date (trial ongoing; mean number of modules completed = 3.1).

Of the control participants, five (71.4%) accessed the BCNA digital resources once, and two (28.6%) accessed them fortnightly, over the study period. This was higher than engagement rates for the overall control sample to date (35% did not access the resources, 41.7% accessed once, 18.3% accessed fortnightly, and 5% accessed weekly).

### Thematic analysis

**Fig. 1:**
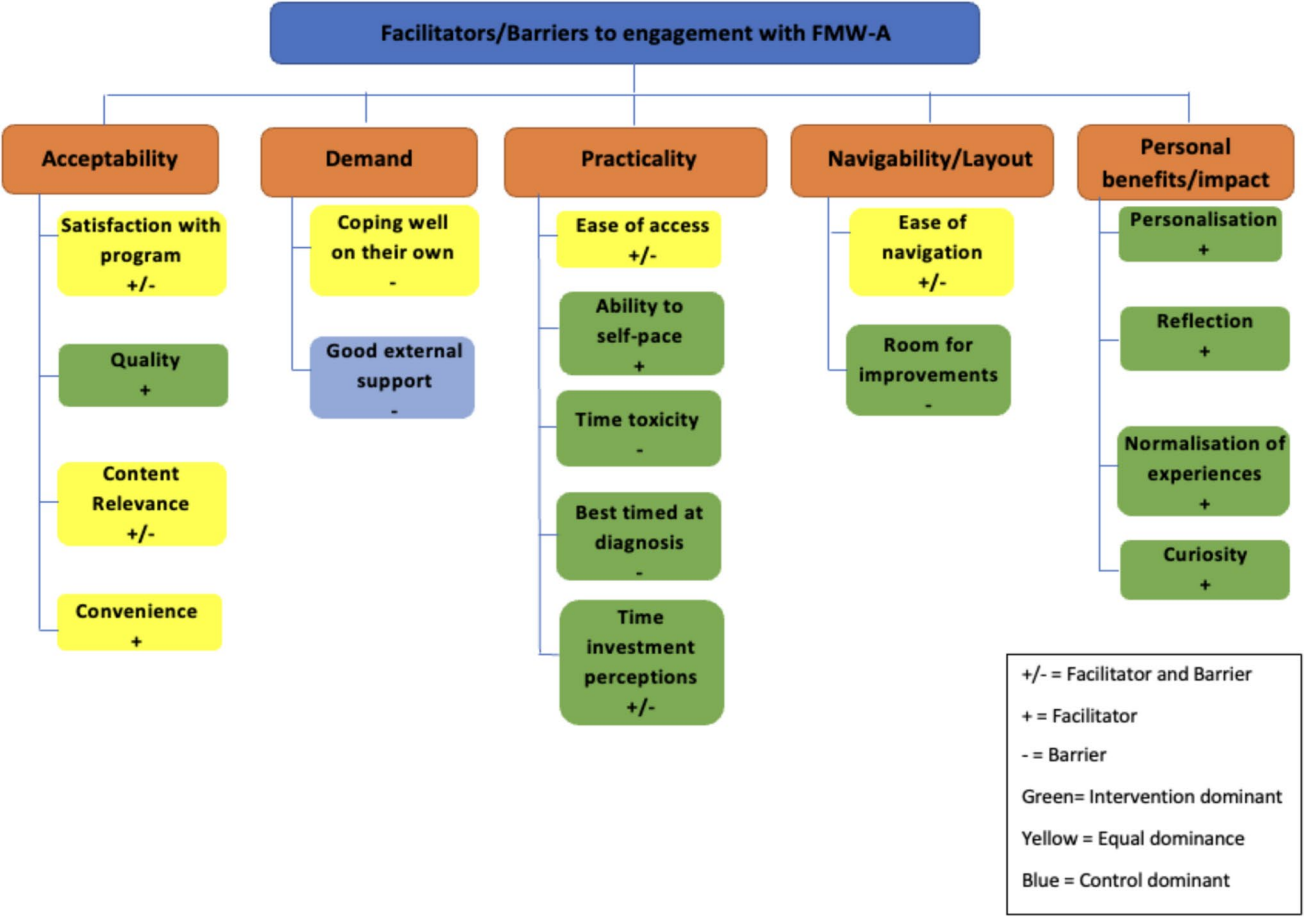
Thematic map

Figure 1 outlines the final thematic map, while Table 3 outlines the coding framework. Five themes emerged from analysis: three were deductively coded using a priori areas from Bowen’s [31] framework (acceptability, demand, and practicality); two were inductively identified (navigability/layout, personal benefits/impact). Each contained up to five subthemes, summarised below and in Table 3 with illustrative quotes. Table 4 summarises facilitators and barriers to engagement across both groups.

**Table 3:** Thematic table, of all themes, subthemes, definitions, and illustrative quotes

Theme/definition	Subtheme (*N* intervention; *N* control)	Codes	Illustrative quote
**Acceptability** *Participants’ judgment and response to the program*	Satisfaction with the program (11/13 intervention; 5/7 control)	Satisfaction with the program Child code: content; enjoyed reading through the information	“The one thing I was thinking of… it might be useful to have information about up-and-coming treatments like immunotherapies and things like that, just things that maybe aren’t absolutely available yet, but are steps in the direction of further treatments and so on.” (Participant 6, Control) “I think it was really good. I think for me there’s an element of … I think some of what I’m looking for, in terms of information…, that I didn’t find there was things like, …, you know, I’m working full time… I’m in my mid-40 s, I’ve got older kids… there’s some things specific to where I’m at in my stage of life that I’m looking for that I don’t think I necessarily found in there…I mean you can’t cover everybody, but that’s yeah that’s something for me that I’m looking for right now.” (Participant 7, Intervention, high-engager)
	Quality (9/13 intervention; 1/7 control)	Quality Child code: good quality; easy to read through the content; trustworthy/credible	“Yeah quality, quality is good, you have a number of people who are just out of the general population and then you have a couple of well-studied people and I probably find that their information is good because, the others are really talking about their experiences. And that too is valuable but it’s actually nice to have expert stuff happening…if this is applicable to you then maybe look at this. So that was good.” (Participant 12, Intervention, high-engager)
	Content relevance (4/13 intervention; 2/7 control)	Relevance Child code: information was relevant; irrelevant	“I think it really depends on what the individual’s needs are. So … I would understand that there will be people who will want to engage with all aspects of it and really … you know develop their knowledge and their personal skills, … for me, … I don’t focus on my cancer, …, I guess I didn’t find it as useful as I might have, I might when I’m potentially further down the track. Uhm and you know experiencing more uhm you know side effects and impacts from it, so I think my analysis of it is that I think it is a useful program, but I think it’s about what people need at the point of time they’re in, the context they’re in and then enabling, … having the information that people might want to dip in to.” (Participant 15, Intervention, low-engager) “I did intend to use it more, but also I just kept finding that there wasn’t that much that was relevant for me, so I’d look at a few things or look back at the same thing…” (Participant 17, Control)
	Convenience (7/13 intervention; 5/7 control)	Convenience	“Really easy once you logged in its just there for you, it’s really easy.” (Participant 10, Intervention participant, high-engager) “You can use it whenever its convenient for you.” (Participant 9, Control)
**Demand** *The perceived need for the program (either as individuals, or for the population more broadly)*	Coping well on their own (2/13 intervention; 2/7 control)	Coping well on their own	“… I don’t know whether I’m just living in denial or something at the moment but, I don’t think I am. But I don’t sort of feel the need for you know the extra help that could be available such as psychologists. You know and all those sorts of extra things. I think my outlook is pretty positive and I think I’m fairly realist when it comes to later stages of the disease, I’m not really denying that this is going to happen, but I think I live my life on a, on a more upbeat note than some of those modules suggested that I should be…” (Participant 11, Intervention, low-engager) “Yeah but I’m having a pretty good time with things and not a lot has changed for me in the last sort of 7 years except for change in treatments, but you know that’s going okay, and I’m well.” (Participant 17, Control) “I kind of looked at it and thought I don’t really see that there is much there that is of interest to me. You know I’ve been lucky I haven’t needed things like Beyond Blue and that sort of thing, and my oncologist is pretty good at answering questions if I have them. I mean, I do get that a lot of people need support, but I’m one of those people that tends to sort of want to do it my way.” (Participant 6, Control)
	Good external support (1/13 intervention; 2/7 control)	Good external support	“… I did expect to use it a lot and I guess that’s one of the things is … I think probably for where I was at the time that I started it I was also starting work with a psychologist so they probably helped me in some spaces and meant that I didn’t delve into the program as much because I was working with her” (Participant 7, Intervention, high-engager) “I’ve been very lucky, I’ve got support around me and that sort of thing, I’ve not had lots of bad times, and it’s just been I’ve been lucky” (Participant 6, Control)
**Practicality** *The extent to which the intervention could be used when time, resources, external factors, or a combination of these were restricted in some way*	Ease of access (3/13 intervention; 2/7 control)	Easily accessible	“I really like the fact that I’ve got the information to keep going back to.” (Participant 16, Intervention, high-engager) “It’s very clear about all the different sections that are available to engage with, in terms of its structure, it has good clarity.” (Participant 3, Control)
	Self-pace (6/13 intervention; 0/7 control)	Self-pace was useful	“If I didn’t want to finish that module, I didn’t have to so I could go out and then come back in later.” (Participant 10, Intervention, high-engager)
	Time burden—competing demands (5/13 intervention; 4/7 control)	Barriers to use: Child code: personal: life got in the way, other priorities; interpersonal: family circumstances	“I would start to do something or read through something and then I’d get interrupted. And it’s like my situation, my life. I did find that annoying, because I would like to just sit down and just do it but just the way things are in my life. You know my husband and I run a business, … and I’ve got three kids and a grandchild, and one daughter is pregnant… so it’s a busy busy, my parents and my mother-in-law, so it’s a very busy time for me, … I did find having it on my phone was good but then I’d start it.” (Participant 1, Intervention, low-engager) “… not really. I think my life circumstances have been pretty full on. And that’s taken up a lot of time, and energy just dealing with aging parents and dying parents …” (Participant 3, Control participant)
	Time burden—health-related barriers (9/13 intervention; 5/7 control)	Health-related Child code: busy with treatment, feeling fatigued, health issues, needed to attend doctor’s appointment, psychological, treatment side effects	“I was having treatment, … other doctors’ appointments, … and because I’m having some of that treatment, I am finding myself very fatigued. So, I think that was probably the main reason I didn’t get to it. It wasn’t because I didn’t want to you know address anything. It was more of a time thing for me.” (Participant 1, Intervention, low-engager) “There were times that I just didn’t feel like having anything related to cancer so I tried not to do it all the time every day.” (Participant 4, Intervention, low-engager)
	Time investment perceptions (6/13 intervention; 0/7 control)	Time commitment; time-consuming; time required to work through was acceptable	“Maybe a little bit long some of them, I can’t exactly recall which one. But it felt like maybe it was the ones like the Death and dying section. You just didn’t really want to dwell on it for too long.” (Participant 2, Intervention, low-engager)
	Best timed at diagnosis (6/13 intervention; 0/7 control)	Timing of the intervention	“My diagnosis of metastatic cancer is now four years ago so I’ve been living with cancer for four years, and in fact living with cancer from my first diagnosis for 7 years, … I think I was, I felt more vulnerable and in need of information at the very beginning when I first had been diagnosed, and I think it would have been timely then.” (Participant 15, Intervention, low-engager)
**Navigability/layout** *Participants’ ability to easily navigate through the program/resources, and their experiences with the layout and structure of the program/resources*	Ease of navigation (8/13 intervention; 5/7 control)	Interface and structure were good and easy to navigate	“Certainly, having it grouped in the modules was a, is a nice way because you can then … dip in to a particular module and it’s discreet and you can identify something that … you might feel like there’s a particular need for it at a given point in time.” (Participant 18, Intervention, high-engager) “…It was easy I thought it was very well designed actually. I liked the way it links through and you can go back and … you know its nicely set. I think it’s a nice design.” (Participant 17, Control)
	Room for improvements (4/13 intervention; 0/7 control)	Ways to improve program and overall usage	“Look … there might be a slight for me when I want to learn something I probably need … a bit of a search it factor. Say for example … side effects of the medication, if I wanted that, if I can put it in and then find that info within the program. Cause I read something, and I haven’t been able to come across it since so I’m not sure if I made it up or … it was something else that I was working you know looking at the time. I remember doing some sort of questionnaire about your shakiness and I haven’t been able to find that since. So, … some sort of search within the module would be handy.” (Participant 12, Intervention, high-engager) “It would be good to I think if you could have an app version of it so that you can use it on your phone when you’re like in treatment if you don’t have a tablet sort of thing. Making it more accessible on the phone would probably be the only thing that I would make it easier for me…” (Participant 4, Intervention, low-engager)
**Personal benefits/impact** *The personal benefits and impact that participant’s felt that the intervention offered them*	Personalisation (4/13 intervention; 0/7 control)	Personalisation Child code: able to access preferred modules; focused on personal interests; ability to personalise the way they engaged with the program	“I did it in bits and bats which is why I ended up using a book because I often ended up doing it when I didn’t have good internet. So, I started writing a journal with it.” (Participant 16, Intervention, high-engager)
	Reflection (7/13 intervention; 0/7 control)	Allowed reflection Child code: allowed them to think about their situation and future; brought up new questions; made them think about what they need	“It helped because it made you think a little bit about what’s going on in your own situation.” (Participant 1, Intervention, low-engager) “… I can honestly say it did start some conversations. I don’t know if it was the questionnaire at the start or the questionnaire that you keep doing on your mental health type thing. But it did start some questions between myself and my husband, … in regards to you know, like if the inevitable happened and I go into palliative care and need to be treated and stuff, so … a few years ago my stepsister uhm passed away with bowel cancer. But she passed away at home and I was the one that looked after her. So it sort of started that conversation between me and my husband of what would happen if we got to that point. Which yeah, one of the questionnaires sort of hit on a little bit, so it was good in that respect.” (Participant 5, Intervention, low-engager)
	Normalisation of experiences (1/13 intervention; 1/7 control)	Normalisation	“… I thought that was good because it you know puts you in the realm of not being alone that there are hundreds of people facing the same thing.” (Participant 11, Intervention, low-engager) “I think seeking other peoples’ … experiences just gives a really good context. … cause its new for me. And … just reading people’s living with metastasised cancer for years and … also validating how it feels, that the rollercoaster ride the emotional rollercoaster that it is. It was very validating in that regard, reading other people’s stories and that validating my own experiences around my emotional response to everything.” (Participant 3, Control)
	Curiosity (3/13 intervention; 1/7 control)	Curiosity Thirst for information	“I think, there’s just like a thirst for information. … it’s hard to sort of know where to go especially I think when you’re newly diagnosed, you’re just, I don’t know, I am the sort of person that just wants to know more and more.” (Participant 7, Intervention, high-engager)

**Table 4:** Summary of facilitators and barriers to engagement

Facilitators	Barriers
Satisfaction with program	Difficulties with accessibility and navigation
**Quality of the program**	Irrelevant content
Relevance of the program	Perceived lack of need due to coping well on their own or having good external support
Convenience and ease of navigating through the program	**Technical barriers**
**Ability to self-pace**	Experiencing time burden
**Personal benefits or impact**	**High time investment required**
	**Optimal timing of intervention**
	**Room for improvements**

Bolded items relate to factors which were predominantly or solely reported by FMW-A participants

#### Theme 1: Acceptability

Acceptability was defined as participants’ judgment and response to the program. Subthemes included satisfaction, quality, content relevance, and convenience.

*Satisfaction* included participants’ satisfaction with program *content*, discussed by both groups. Within this subtheme, one dominant facilitator and two barriers were raised: The facilitator (discussed by both groups) was that content was *easy to read and understand* (11/13 intervention; 5/7 control). One barrier was reported by intervention participants only, that program content brought up “uncomfortable feelings” (7/13 (of whom 53.8% were high-engagers)), often linked to confronting content, e.g., mortality. A second barrier (reported proportionally more frequently by control participants) was that information was missing (i.e., insufficient input from experts; information related to stage of life, physical aspects of the disease (3/13 intervention; 4/7 control)).

*Quality of the program*, including information provided and overall program quality, was discussed predominantly by intervention participants and reported as a facilitator. Specifically, nine intervention participants (vs one control participant) commented on quality of their allocated resource. One intervention participant commented on the credibility of the program, which positively influenced their perceptions. Another found the expert input and lived-experience stories of women with MBC beneficial. (See Table 3).

*Content relevance*, reported equally by both groups, was discussed as both a barrier and facilitator but raised as a facilitator more frequently. Specifically, six participants reported the information was relevant to their MBC experience (4/13 intervention (75% high-engagers); 2/7 control). Contrastingly, four reported some information was irrelevant to their needs (2/13 intervention (one high-engager); 2/7 control).

*Convenience* of using the program was a facilitator, reported by both groups (7/13 intervention (85.7% high-engagers); 5/7 control).

#### Theme 2: Demand

Demand was defined as perceived need for the program (for either individual participants or the population more broadly). Only *barriers* to engagement emerged, with subthemes relating to participants having *low demand* for the program due to coping well on their own and having good external support. This barrier emerged equally across the two groups.

*Coping well on their own* due to either feeling physically well or having prior knowledge of cancer was reported by four participants (2/13 intervention (one low-engager); 2/7 control). One low-engager intervention participant specifically reported prior knowledge; they had first been diagnosed over a decade ago and already had ample knowledge. Similarly, another participant felt they were coping well and did not need additional support.

*Good external support*, through either healthcare teams or personal supports, was reported by three participants (1/13 intervention (high-engager); 2/7 control), who noted this reduced their perceived need for online programs.

#### Theme 3: Practicality

Practicality was defined as the extent to which the intervention could be used when time, resources, external factors, or a combination, were restricted. Subthemes included* ease of access*, *ability to self-pace*, *time burden* including whether the required *time investment was perceived as worthwhile*, and *optimal timing*.

*Ease of access *to the program was both a facilitator and barrier to engagement reported by both groups. Specifically, five participants reported the program/resources were easily accessible (3/13 intervention (100% high-engagers); 2/7 control). Two intervention participants, both high-engagers, reported ease of access could have been enhanced by having an app version of the program.

In contrast, one barrier identified by intervention participants only, emerged regarding technical barriers to accessing the program on phone or tablet or logging in (8/13 intervention (62.5% high-engagers); 0/7 control).

*Ability to self-pace* was a facilitator reported only by intervention participants. Specifically, six (83.3% high-engagers) reported the self-paced nature of the program was useful.

*Time burden* was raised as a barrier to engagement, in combination with external factors that reduced participants’ available time (i.e., competing demands and health-related barriers).

Competing demands in terms of personal and interpersonal factors were identified as reducing available time to engage. Personal factors (4/13 intervention (100% low-engagers); 3/7 control) included work commitments, general sense of “life getting in the way,” and other health priorities, i.e., healthy diet and being active (reported by two control participants). Interpersonal factors (1/13 intervention (high-engager); 1/7 control) included family circumstances impacting on availability to fully engage (e.g., a family member passing away, looking after an ill relative).

Health-related barriers were reported to interact with time-availability barriers to reduce engagement across both groups but were particularly salient among intervention participants. These included being busy with medical treatment (1/13 intervention (low-engager); 1/7 control), attending doctor’s appointments (2/13 intervention (100% low-engagers); 1/7 control), feeling fatigued (1/7 control), managing health-related complications (1/13 intervention (high-engager)) or treatment side effects (1/13 intervention (low-engager)), and psychological factors such as needing the right mindset or avoidance (3/13 intervention (67% high-engagers); 1/7 control). However, six participants reported no time burden barriers (4/13 intervention (100% high-engagers); 2/7 control).

*Time investment perceptions* were raised as both a barrier and facilitator by intervention participants only. Specifically, two (high-engager) participants reported the time required to complete the program was acceptable. Contrastingly, four participants (75% low-engagers) thought the program was too time-consuming, due to lengthy modules requiring substantial time to complete. Three of these participants added they were personally time-poor (due to time burdens described above) which impacted their engagement.

*Optimal timing -* Best timed at diagnosis*.* One theme predominantly reported by intervention participants (6/13 intervention (67% high-engagers); 1/7 control) was that the intervention would be best-timed at diagnosis. Specifically, some reported the program would have been more useful at first early-stage cancer diagnosis (2/6), while others reported they would have found it more useful at initial MBC diagnosis (4/6). These participants were a mean of 33 months since MBC diagnosis (range = 15–66 months).

#### Theme 4: Navigability/layout

This was defined as participants’ ability to easily navigate the program/resources, and experiences with layout and structure, with both barriers and facilitators emerging. Subthemes included *ease of navigation* and *room for improvements*.

*Ease of navigation* was reported by both groups as a facilitator and barrier. Ten participants reported they found the program/resources easy to navigate, including structure and layout (5/13 intervention (80% high-engagers); 5/7 control). In contrast, three interventions (66.7% low-engagers) reported program structure “could have been simplified.”

*Room for improvements*. Four participants (all intervention participants; 75% low-engagers) suggested possible improvements to navigation and layout that could facilitate engagement. Two suggested a search function; one (high-engager) suggested reducing content volume to ease navigation and time burden.

#### Theme 5: Personal benefits/impact

This theme related to the impact that deriving personal benefits from the intervention had on engagement (i.e., participants could see it was benefitting them, thus wanted to engage more). These were primarily reported by intervention participants (9/13 intervention (55.5% high-engagers); 2/7 control). Subthemes identified included *personalisation*, *reflection*, *normalisation*, and *curiosity*. One regionally residing participant also reported that engaging with the program offered a “sense of connection,” by reducing their sense of isolation (Intervention, high-engager).

*Personalisation:* Four intervention participants (75% high-engagers) reported valuing being able to personalise the way they used the program, e.g., ability to focus on areas of particular interest/relevance to them, or accessing preferred modules first.

*Reflection:* Seven intervention participants (57.1% high-engagers) reported the program enabled them to self-reflect. This theme occurred consistently, including for low-engagers.

*Normalisation of experiences:* One participant from each group reported using the program/resources helped normalise and validate their cancer experiences.

*Curiosity:* Four participants reported curiosity, including thirst for information, led to them engaging with the program/resources (3/13 intervention (66.7% high-engagers); 1/7 control).

## Discussion

This study explored barriers and facilitators to engagement with an interactive DMHI for MBC versus a digital information-resource control-group. Across groups, five broad themes (acceptability, demand, practicality, navigability/layout, and personal benefit/impact) yielded seven facilitators, five barriers, and five factors that acted as both barriers and facilitators. Consistent with prior research (24, 27, 28), program engagement in the current study was facilitated by (i) *satisfaction with the program*, (ii) *perceived quality and relevance of the program’s content*, (iii) *convenience* and *ease of navigating* through the program, and (iv) *ability to self-pace*. Of note, several factors reported as facilitators for some participants were reported as barriers for others, i.e., difficulties with accessibility and navigation, and irrelevant content. This discrepancy confirms prior findings and highlights the importance of tailoring interventions, given what is perceived as easy and relevant for one person may be challenging for another (34).

Factors in the present study likely to act as barriers to intervention engagement were (i) perceived lack of need due to *coping well on their own*, (ii) *technical barriers*, (iii) experiencing *time burden*, and the corresponding perceived *high time investment* required. While these have previously been consistently reported in the literature (24, 25, 35), this is the first study to document their particular salience for metastatic cancer survivors, where time (as a limited, finite, and precious resource) appeared frequently as a lens that participants viewed their experiences of FMW-A through. This fits with recent literature on patient’s time perceptions and time toxicity in informing treatment decision-making (36, 37). Taken together, these facilitators and barriers provide useful guidelines for healthcare providers and researchers, suggesting that DMHI for MBC must be optimally timed, brief, easy to access and navigate, relevant, searchable, and personalisable, to be viewed as a valuable time investment. However, these findings must be qualified by the high overall levels of engagement observed in the qualitative participants, compared with their clinical trial counterparts. How well these findings generalise to low-engagers must be considered with caution.

A key strength of this study was the inclusion of control participants who received general online informational resources, in contrast to intervention participants who received more extensive tailored and interactive psychosocial support. Very few prior qualitative studies have undertaken this comparison, and of those that have, the comparison has been with a study-specific psychoeducational attention-control rather than publicly available online information (24) thus reducing the generalisability of insights that can be drawn. The current study enabled evaluation of differences in barriers and facilitators of engagement between groups, to help differentiate and inform general issues arising when accessing any cancer online resource, versus more specific barriers to be addressed for digital mental health interventions. That is, the themes that were raised equally by both intervention and control groups, or solely by control participants, were more indicative of general issues with any form of digital engagement, whereas themes that were dominantly or solely raised by intervention participants were more specific challenges to interacting with DMHI. While barriers raised by both groups ideally need to be addressed, it is important to distinguish and address the specific additional barriers/burden generated from DMHI to maximise their feasibility and impact. One key difference identified between groups was *time investment* as both a barrier and facilitator, reported only by intervention participants. Given that the *FMW-A* program was larger (with practical tasks and activities to complete) than the *My Journey* control resource, it had an objectively larger time commitment required. Whether this commitment was viewed as a valuable investment or a burden appeared to differentiate engagement levels; three of four participants who reported the program was time-consuming (i.e., the commitment was not worthwhile) were low-engagers. In contrast, the two participants who found the time commitment involved was acceptable (i.e., perceived the time investment as worthwhile) were high-engagers. Prior research has shown that the length and pace of online self-guided CBT programs can impact user motivation (38), and participants report a preference for more concise modules (39). Thus, it is likely that participants who reported finding the *FMW-A* program too time-consuming experienced motivation reductions, and consequently engagement. In contrast, given the objectively smaller time commitment required for the control-group, these perceived time trade-offs (investment vs burden) were not evident.

Another facilitator observed only among intervention participants was the ability to self-pace, similar to Beatty et al.’s (24) findings involving the original *FMW* program. Prior research highlights the importance of self-pacing when developing online interventions (26). Although unclear why self-pacing was only mentioned by intervention participants, it is possible that self-pacing only becomes salient when there is a greater amount of content to digest, as occurred in the *FMW-A* program compared to control resources. Self-pacing is evidently an important aspect of online interventions, with research demonstrating it fosters a sense of empowerment over one’s healthcare needs (40).

Interestingly, despite quantitative evidence that the intervention and control resources were taken up and used by participants, qualitatively, they did not cite a demand for these resources, and instead, only *barriers* to engagement emerged (i.e., low demand due to coping well on their own, having good external support). These findings could partly be explained by the other factor predominantly identified as a barrier by intervention participants, that the intervention would be most optimally timed (and beneficial) at diagnosis. This finding has been previously raised in the literature (8) and suggests that demand may have dropped for some participants due to having their needs met elsewhere. Although it did not hinder overall engagement levels, these findings are important to address. Six participants reported it would have been more beneficial to have accessed the program at the time of diagnosis (either first early-stage cancer diagnosis and/or MBC diagnosis). Given that research indicates early intervention is linked with better outcomes (41), further exploration would be beneficial to establish optimal timing for offering the program. This could potentially be achieved via integration into optimal care pathways, where all individuals with cancer are routinely screened for symptoms and unmet needs throughout the cancer trajectory and referred to DMHI programs when appropriate (42, 43). This would enable analysis of the optimal timing of the intervention.

Importantly, through inductive analysis, a new theme was developed, i.e., the impact of deriving *personal benefits* on subsequent engagement (44). This was predominantly reported by intervention participants (i.e., 69% vs 28.5% for control). While more associated with high engagement, even those with relatively low engagement still reported some personal benefits/impact from participation. Whether this qualitative finding corresponds with being an early-responder to treatment would be an interesting avenue for future analysis.

While this study has yielded novel insights, they must be considered in light of the two key limitations. First, the high proportion of high-engagers in the intervention group limits the range, and generalisability, of potential barriers that could be identified. Additional barriers may exist for low-engagers beyond those identified in the current study; the broader DMHI literature suggests these might include socioeconomic barriers (for example, older age, experiencing access barriers due to socioeconomic disadvantage, or reduced relevance for specific cultural groups), digital literacy, mistrust of online programs, or experiencing severe mental health issues (35, 45, 46). Further, evidence shows that people are likely to engage more in clinical trials compared to real-world interventions/resources; thus, current findings may underestimate barriers and/or facilitators experienced outside of clinical trials (47, 48). Future studies could aim to specifically recruit low-engagers and/or in real-world, non-clinical trial settings for qualitative follow-up. Second, the study was constrained by the sociodemographic characteristics of the sample: Caucasian, English-speaking, and well-educated. It is likely difficult to generalise these findings to people from other culturally or linguistically diverse groups, and further research involving more diverse samples is necessary. Strategies to address this need to start at study inception and should be integrated throughout the research pipeline, via (i) co-designing the interventions with members from culturally and linguistically diverse groups to ensure content relevance and sensitivity and (ii) partnering with relevant clinical, consumer and community organisations, who are then positioned to champion the DMHI resources with their members. These strategies have been shown to increase the perceived credibility of, and trust in, developed programs (49).

In conclusion, this study confirmed the applicability of known factors that facilitate and impede engagement with online interventions (24, 25, 28, 35) to the metastatic setting, documented a key new facilitator—deriving early personal benefit—and emphasised the need for web-programs to be timed at diagnosis.

## Data Availability

Data is provided within the manuscript or supplementary information files.
